# Bacterial Etiology and Risk Factors Associated with Cellulitis and Purulent Skin Abscesses in Military Trainees

**DOI:** 10.1371/journal.pone.0165491

**Published:** 2016-10-25

**Authors:** Ryan C. Johnson, Michael W. Ellis, Carey D. Schlett, Eugene V. Millar, Patrick T. LaBreck, Deepika Mor, Emad M. Elassal, Jeffrey B. Lanier, Cassie L. Redden, Tianyuan Cui, Nimfa Teneza-Mora, Danett K. Bishop, Eric R. Hall, Kimberly A. Bishop-Lilly, D. Scott Merrell

**Affiliations:** 1 Department of Microbiology and Immunology, Uniformed Services University of the Health Sciences, Bethesda, Maryland, United States of America; 2 The University of Toledo Medical Center, Toledo, Ohio, United States of America; 3 Infectious Disease Clinical Research Program, Department of Preventive Medicine and Biometrics, Uniformed Services University of the Health Sciences, Bethesda, Maryland, United States of America; 4 Martin Army Community Hospital, Fort Benning, Georgia, United States of America; 5 The Henry M. Jackson Foundation for the Advancement of Military Medicine, Inc, Bethesda, Maryland, United States of America; 6 Biological Defense Research Directorate, Naval Medical Research Center, Frederick, Fort Detrick, Maryland, United States of America; 7 Infectious Diseases Directorate, Wound Infections Department, Naval Medical Research Center, Silver Spring, Maryland, United States of America; 8 Department of Medicine, Uniformed Services University of the Health Sciences, Bethesda, Maryland, United States of America; Purdue University, UNITED STATES

## Abstract

Military trainees are at high risk for skin and soft-tissue infections (SSTIs). Although *Staphylococcus aureus* is associated with purulent SSTI, it is unclear to what degree this pathogen causes nonpurulent cellulitis. To inform effective prevention strategies and to provide novel insights into SSTI pathogenesis, we aimed to determine the etiology of SSTI in this population. We conducted a prospective observational study in US Army Infantry trainees with SSTI (cutaneous abscesses and cellulitis) from July 2012 through December 2014. We used standard microbiology, serology, and high-throughput sequencing to determine the etiology of SSTI. Furthermore, we compared purported risk factors as well as anatomic site colonization for *S*. *aureus*. Among 201 SSTI cases evaluated for SSTI risk factors, cellulitis was associated with lower extremity blisters (P = 0.01) and abscess was associated with methicillin-resistant *S*. *aureus* (MRSA) colonization (P<0.001). Among the 22 tested cellulitis cases that were part of the microbiome analysis, only 1 leading edge aspirate was culturable (Coagulase-negative *Staphylococcus*). Microbiome evaluation of aspirate specimens demonstrated that *Rhodanobacter terrae* was the most abundant species (66.8% average abundance), while abscesses were dominated by *S*. *aureus* (92.9% average abundance). Although abscesses and cellulitis share the spectrum of clinical SSTI, the bacterial etiologies as determined by current technology appear distinct. Furthermore, the presence of atypical bacteria within cellulitis aspirates may indicate novel mechanisms of cellulitis pathogenesis.

Clinical Trials Registration: NCT01105767.

## Introduction

Skin and soft-tissue infections (SSTIs) are among the most frequently observed infections in the ambulatory and hospital settings [1–3]. In 2005 alone, approximately 14.2 million SSTI cases were reported in the United States; a 65% increase from 1997 [2]. Although severe SSTIs such as necrotizing fasciitis are encountered, the bulk of disease manifests as abscesses and cellulitis [2]. Despite numerous established SSTI risk factors, whether certain risk factors are specifically associated with either cutaneous abscess or cellulitis formation have yet to be determined [4].

Although SSTIs afflict millions of individuals world-wide, certain congregate populations, such as military service members, are at increased risk [5, 6]. While *Staphylococcus aureus*, and in particular community-associated methicillin-resistant *Staphylococcus aureus* (CA-MRSA) has been identified as the leading cause of most purulent SSTIs [7, 8], to what extent this pathogen causes nonpurulent cellulitis remains unknown.

Without a site to culture, bacterial etiology for nonpurulent cellulitis has been historically unsuccessful. Dogma suggests that cellulitis is caused by both *S*. *aureus* and beta-hemolytic streptococci (BHS); however, the actual degree each pathogen contributes is unclear [9–11]. Additionally, despite diverse sample collection methods (punch biopsy, needle aspirate and blood sample), a pathogen is isolated less than 30% of the time [12–17]. The changing epidemiology of SSTI and uncertainty of microbial etiology of nonpurulent cellulitis have exposed important clinical knowledge gaps.

Herein we report our findings from a prospective observational study aimed at discerning SSTI-specific risk factors as well as detailed characterization of the microbiology of both purulent and nonpurulent SSTI using a high-throughput sequencing strategy in addition to standard microbiology and serology. Results suggest that atypical bacteria may play an important role in nonpurulent cellulitis pathogenesis.

## Materials and Methods

### Study design

This was a prospective observational study using clinical data, anatomic site colonization information, acute and convalescent serum specimens, risk factor questionnaires, and leading edge aspirate or abscess specimens to determine the etiology of cutaneous abscesses and nonpurulent cellulitis and to determine risk factors associated with each SSTI manifestation. The study population consisted of US Army soldiers undergoing 14-week Infantry training at Fort Benning, Georgia. This all male population was ethnically diverse, in good general health, and between the ages of 17 and 42 years. As defined in DoD Directive (DoDD) 3216.02 “For purposes of legal capacity to participate in DoD-conducted or -supported research involving human subjects, all active duty Service members and all Reserve Component members in a Federal duty status are considered for purposes of this Instruction to be adults.” Thus, any individuals that were 17 years of age were consented as adults. The Uniformed Services University Infectious Diseases Institutional Review Board approved the investigation and written consent procedure (IDCRP-074). All participants provided written consent to participate in the study.

### Eligibility, Enrollment, and Data Collection

We identified potential participants and enrolled them prospectively. Eligible participants consisted of Infantry trainees seeking medical care at the Troop Medical Clinic (TMC) for SSTI from July 2012 to December 2014. All eligible trainees were approached and consented to allow investigators to: 1) obtain pertinent SSTI-related information from the electronic medical record, and 2) to obtain pertinent specimens from the clinical microbiological lab. At enrollment, all consenting participants underwent anatomic site (anterior nares, oropharynx, inguinal, and perirectal) screening for *S*. *aureus* (BD BBL CultureSwabs® (BD Diagnostic, Sparks, MD)) and completed a questionnaire to assess prior exposure to SSTI risk factors [18].

We defined cellulitis as an acute infection characterized by diffuse inflammation of skin and associated skin structures with at least two of the following signs or symptoms on the affected area: erythema, edema or induration, warmth, tenderness to palpation or pain. We defined abscess as an acute skin and skin structure infection characterized by a collection of pus within the dermis or deeper that was accompanied by erythema, edema, and/or induration, and tenderness. We defined nonpurulent cellulitis as cellulitis with no purulent drainage or exudate and no associated abscess. For inclusion as a nonpurulent cellulitis participant, the minimum affected area of erythema, edema, and/or induration was 36 cm^2^. Participants were consented and enrolled independent of abscess/cellulitis body location except as indicated below. All obtained cellulitis aspirates were included in subsequent microbiome analyses and comparable numbers of abscess samples were randomly selected for inclusion.

Exclusion criteria included prior antibiotic administration within two weeks before initial presentation, infection on the face or associated with a surgical site, animal or human bite wound, evidence of suspected bacteremia/sepsis/deep soft tissue infection, or any underlying condition such as neutropenia, vascular insufficiency, or diabetes.

### Study procedures

For participants with abscesses, the infection site was cleaned with 4% chlorhexidine and isopropyl alcohol prior to incision. Two swabs (BD BBL CultureSwabs® (BD Diagnostic, Sparks, MD)) were then obtained from within the abscess cavity: one was sent for microbiological culture, and the other was immediately placed at -80 C until used for microbiome analysis. For participants with nonpurulent cellulitis, the leading edge aspirate was performed in sterile fashion in the following manner. After the leading edge of maximum inflammation was identified, the area was first anesthetized using occlusive dressing and topical lidocaine-prilocaine (Emla ™ cream), then cleaned with 4% chlorhexidine solution and isopropyl alcohol wipe. A 22-guage needle attached to a 10 ml sterile syringe loaded with 1.5 ml sterile nonbacteriostatic saline was introduced into the subcutaneous tissue, and the solution was slowly injected forming a wheal, then immediately aspirated. One half of the recovered volume (approximately 0.5 ml) was immediately frozen at -80 C and sent for microbiome analysis. The remaining volume (approximately 0.5 mL) was immediately inoculated into 10 ml of fastidious broth (Remel, Lenexa, KS) and processed for bacterial culture. Post aspiration, the participant underwent phlebotomy to obtain serum samples for immunological surveillance (acute samples) and was monitored for 21–28 days at which time a second phlebotomy was performed (convalescent samples). Follow-up assessments were done at post-procedure days 2–3 and 21–28 for all participants to monitor SSTI resolution.

Anatomical site colonization samples were cultured in 6.5% NaCl enriched broth as previously described [19]. Leading edge aspirate specimens were incubated at 35°C in CO2. Specimens were assessed daily for visible turbidity; and when present, were subcultured onto TSA 5% Sheep blood, MacConkey, chocolate, and anaerobic plates using standard lab techniques. After five days of incubation, blind subculture was performed in the same manner. Abscess cultures were conducted according to standard procedures at the Martin Army Community Hospital microbiology laboratory [20]. *S*. *aureus* isolates were typed using pulsed-field gel electrophoresis (PFGE) and resistance/virulence determinants were assessed using PCR as previously described [21].

### Serological analysis

Acute and convalescent blood samples were allowed to clot for a minimum of 30 minutes and serum was isolated by centrifugation at 2500 rpm for 15 minutes. Anti-streptococcal DNase B, and Streptolysin O (ASO) antibody titer determinations were performed at the reference laboratory QuestDiagnostics (Chantilly, VA).

### DNA extraction, amplification, sequencing, and statistical analysis

For abscess swabs, bacterial genomic DNA extraction and PCR amplification was performed in an identical fashion as previously described [18]. For the cellulitis aspirates, 0.5 mls of the recovered nonbacteriostatic saline was mixed with an equal volume of lysis solution (GenElute bacterial genomic DNA kit (Sigma-Aldrich)) containing lysozyme (45 mg/ml), mutanolysin (125 U/ml), and lysostaphin (0.16 mg/ml) and allowed to incubate for 30 min at 37°C. Proteinase K (0.95 mg/ml) and lysis solution C (1 ml) were subsequently added to the sample and incubated for 10 minutes at 55°C. The remaining column purification steps were performed to the manufacturer’s specifications. Amplification of the V1-V3 region of the 16S rRNA gene from the purulent abscess and cellulitis specimens was performed as previously described [18]. All samples were multiplexed and sequenced in a single 454 pyrosequencing run using the Roche GS FLX Titanium 454 sequencer at the Naval Medical Research Center in Fredrick, Maryland. Raw DNA sequences were quality processed and assigned taxonomy information using Mothur (v.1.34.4) as previously described [18]. Subsampling was not performed for our data set given that no diversity comparisons were utilized. To control for possible reagent contaminates, we also prepared 0.5 mls of sterile nonbacteriostatic saline (the same as used for aspirate collection) for 16S amplification and sequencing using the same reagents and 454 sequencer as described above. The nonbacteriostatic saline sample was sequenced in a separate pyroseqeuncing reaction in combination with an ongoing fecal microbiome study in our laboratory and yielded 3 usable reads; all of which were assigned to the alphaproteobacteria class (S1 File).

For full-length 16S rRNA gene sequencing, total genomic DNA from four randomly chosen cellulitis samples were subjected to PCR amplification of the 16S rRNA gene using the 8F and 1492R primers [22]. The PCRs were performed in 50 μl reactions containing nuclease-free water, 125 U FideliTaq DNA polymerase (Affymetrix), and 0.6 μM of the forward and reverse primers. PCR mixtures were incubated at 95°C for 90 seconds, 34 cycles of 95°C for 30 seconds, 50°C for 30 seconds and 68°C for 2 minutes followed by a final extension of 68°C for 5 minutes. Five technical replicates of each PCR reaction were combined and purified using the QIAquick PCR Purification Kit (Qiagen). The purified PCR products were then ligated into the pGEM-T easy vector (Promega) and transformed into *E*. *coli* TOP10 cells. Transformants were selected on LB agar supplemented with Ampicillin (Amp) (100 μg/ml), X-gal (40 μg/ml) and IPTG (1 μM). At least 10 white colonies per transformation were selected and grown overnight in LB-Amp broth with shaking at 200 rpm. Plasmids were isolated using the QIAprep Spin Miniprep Kit (Qiagen).

Seven sequencing reactions were performed for each plasmid sample to ensure complete coverage of both strands of the 16S rRNA gene. The insert within the pGEM-T easy vector was sequenced using the following primers: T7, SP6, 8F, 1492R, 515F, 806R, and 919F (Promega) [22, 23]. Sequencing was performed as previously described [24]. DNA chromatograms were visualized using Chromas Lite (Technelysium). Full-length 16S sequences were manually assembled and taxonomy information was assigned after alignment to the RDP database [25].

### Statistical analyses

For the risk factor assessment analysis, p-values for the “median age, years (range)” and “median (range) no. of weeks in training at enrollment” were calculated with the Wilcoxon rank-sum test. For all other comparisons, the chi square test was used to generate p-values. When variable counts were less than 5, the Fisher’s exact p-value was used.

## Results

### Participant characteristics

We enrolled a total of 241 trainees (121 abscess and 120 cellulitis). Of these, 40 (18 abscess and 22 cellulitis) were part of the microbiome portion of the study and 201 were utilized for SSTI risk factor assessment. Demographic and questionnaire data for the 201 SSTI risk factor assessment participants (103 abscess, 98 cellulitis) are shown in Table 1. Characteristics of the microbiome participants are outlined in Table 2 and Table 3.

**Table 1:** Abscess and cellulitis risk factor assessment.

	SSTI Cases (n = 201)		
	Abscess Only^a^ (n = 103)	Cellulitis Only^a^ (n = 98)	P-Value^b^
**Median Age, years (Range)**	19 (18–21)	20 (18–22)	0.06
**Race/Ethnicity**^**c**^			**0.04**
White, Non-Hispanic	65 (63.1%)	78 (79.6%)	
Hispanic	23 (22.3%)	13 (13.3%)	
Black, Non-Hispanic	11 (10.7%)	3 (3.1%)	
Others, Non-Hispanic	3 (2.9%)	4 (4.1%)	
**Site of Infection**^**d**^			
Lower extremity	49 (47.6%)	75 (76.5%)	**<0.001**
Upper extremity	41 (39.8%)	24 (24.5%)	**0.02**
Head	5 (4.9%)	0 (0%)	**0.03**
Thorax	11 (10.7%)	0 (0%)	**<0.001**
Groin/Inguinal/Perineal	1 (1%)	0 (0%)	1
**Median (Range) No. of weeks in training at enrollment**	5 (3–8)	4 (2–7)	0.24
**Season of Enrollment**			
Spring	21 (20.4%)	22 (22.4%)	0.58
Summer	49 (47.6%)	39 (39.8%)	
Fall	22 (21.4%)	21 (21.4%)	
Winter	11 (10.7%)	16 (16.3%)	
**Nasal Colonization with *S*. *aureus***	54 (52.4%)	50 (51%)	0.84
**Nasal Colonization with MRSA**	24 (44.4%)	7 (14%)	**<0.001**
**Any Site Colonization with *S*. *aureus***^**e**^	94 (91.3%)	85 (86.7%)	0.3
**Any Site Colonization with MRSA**^**e**^	59 (62.8%)	23 (27.1%)	**<0.001**
			
**Risk Factor: Past Year**			
Admitted to a Hospital	5 (4.9%)	4 (4.1%)	0.78
Worked at a Hospital	4 (3.9%)	2 (2%)	0.68
Known or suspected SSTI/MSSA Infection	7 (6.8%)	6 (6.1%)	0.83
Taken an Antibiotic in past 6 months	18 (17.5%)	10 (10.2%)	0.13
			
**Risk Factor: 3 mos. prior to Fort Benning**			
Contact with a Person with Skin Infection	4 (3.9%)	2 (2%)	0.68
			
**Risk Factor: While at Fort Benning**			
History of Skin or Soft Tissue Wound	1 (1%)	3 (3.1%)	0.36
Contact with a Person with Skin Infection	30 (29.1%)	25 (25.5%)	0.54
Abrasions/Cuts	57 (55.3%)	51 (52%)	0.64
Blister	44 (42.7%)	59 (60.2%)	**0.01**
Insect Bite	29 (28.2%)	29 (29.6%)	0.82
**Risk Factor: Trainee Survey**			
Wash Hands or Use Sanitizer	103 (100%)	97 (99%)	0.82
Daily Shower	103 (100%)	98 (100%)	0.82
Share Towels and Clothing	2 (1.9%)	6 (6.1%)	0.16
Share or Borrow Razors	1 (1%)	1 (1%)	1
Wash Towels	102 (99%)	94 (95.9%)	0.14
Wash PT Uniform	103 (100%)	96 (98%)	0.15
Wash ACU^f^	103 (100%)	96 (98%)	0.15
Shave Body Parts Other than Face	17 (16.5%)	18 (18.4%)	0.75
Bunkmate/Battle Buddy had Skin/MRSA Infection	13 (12.6%)	7 (7.1%)	0.19
Sexual Contact in Past 6 mos., Someone with Skin Infection	2 (1.9%)	0 (0%)	0.5

^a^ Unless specified, the numbers in parenthesis correspond to the percentage of total individuals with either abscess or cellulitis.

^b^ P-values calculated with chi square test. For counts less than 5, Fisher’s exact test was used. P-values for median data were generated using the Wilcoxon rank-sum test. Significant p-values (< 0.05) are shown in bold.

^c^ Data unavailable for one abscess participant

^d^ Four abscess patients and one cellulitis patient reported more than one SSTI. Lower extremity includes foot, ankle, shin, calf, and thigh. Upper extremity includes forearm, elbow, and shoulder.

^e^ Body sites tested: anterior nares, oropharynx, inguinal, perianal.

^f^ ACU; army combat uniform

**Table 2:** Purulent abscess patient information.

Patient ID	Age / Race	Site of Infection	Abscess Culture	Body Site Colonization			
				Nose	Oropharynx	Perianal	Inguinal
**1782**	22/White	Hand	ND	CNS	CNS	CNS	MSSA
**1868**	17/White	Elbow	MSSA	MSSA	CNS	MSSA	CNS
**1871**	21/White	Elbow	Other^a^	MRSA	NG	CNS	CNS
**1876**	18/White	Buttock	MRSA	CNS	CNS	MRSA	ND
**1914**	18/White	Foot	ND	CNS	CNS	NC	CNS
**1955**	17/White	Lower Leg	MRSA	CNS	NC	NC	NC
**1957**	17/Asian	Forearm	MRSA	MSSA	MSSA	NC	NC
**1983**	18/White	Chest	ND	CNS	CNS	NC	NC
**2018**	18/White	Foot	MRSA	MSSA	MSSA	CNS	CNS
**2058**	26/White	Axilla	ND	MSSA	CNS	NC	CNS
**2067**	22/White	Lower Leg	ND	MSSA	MSSA	CNS	CNS
**2086**	19/Asian	Forearm	ND	CNS	CNS	NC	NC
**2094**	24/White	Knee	ND	CNS	MSSA	NC	MSSA
**2120**	19/White	Axilla	ND	MSSA	MSSA	NC	CNS
**2152**	19/Other	Axilla	ND	MSSA	ND	NC	NC
**2157**	19/White	Hand	Other^a^	NC	MSSA	NC	NC
**2160**	18/White	Foot	Other^a^	MSSA	CNS	NC	NC
**2202**	18/White	Axilla	MRSA	CNS	CNS	NC	CNS

Abbreviations and symbols: ND, not determined; NG, no growth; CNS, Coagulase-negative *Staphylococcus*; NC, not consented; MSSA, methicillin-sensitive *S*. *aureus*; MRSA, methicillin-resistant *S*. *aureus*

^a^ Indicates non-*S*. *aureus* bacterial growth.

**Table 3:** Cellulitis patient information.

Patient ID	Age / Race	Site of Infection	ASO Titers^a^ (Acute/Conv)	Anti-DNase B Titers^a^ (Acute/Conv)	Aspirate Culture	Area Erythema (initial/days 2-3/days 21–28)^b^	Body Site Colonization				Subsequent abscess formation
							Nose	Oropharynx	Perianal	Inguinal	
**1680**	19/White	Foot	ND	ND	NG	203/144/0	MSSA	MSSA	NC	CNS	N
**1739**	17/White	Lower Leg	9/7	<95 /<95	NG	216/212.5/0	CNS	CNS	CNS	MSSA	N
**1773**	21/White	Forearm	**499**/**483**	<95/<95	NG	228/205/0	CNS	NG	MSSA	CNS	N
**2055**	18/White	Foot	**437**/**423**	<95/102	NG	405/240/0	CNS	CNS	NC	NC	N
**2080**	23/White	Foot	92/**324**	<95/<95	NG	-	MSSA	CNS	MSSA	MSSA	N
**2083**	18/White	Foot	**448**/ND	<95/ND	CNS	48/25/-	MSSA	CNS	MSSA	CNS	ND
**2124**	20/White	Foot	**220**/**206**	<95/<95	NG	115/110/0	MSSA	MSSA	MSSA	MSSA	N
**2128**	22/White	Elbow	52/47	<95/109	NG	228/144/0	MSSA	CNS	CNS	CNS	N
**2129**	22/White	Forearm	100/100	109/<95	NG	225/0/0	CNS	CNS	CNS	CNS	N
**2171**	24/White	Thigh	**439**/129	207/124	NG	238/84/0	MSSA	CNS	MSSA	MSSA	Y
**2172**	19/White	Foot	119/113	<95/<95	NG	115.5/116/0	MSSA	MSSA	CNS	CNS	N
**2203**	21/White	Foot	41/37	97/111	NG	-	MSSA	NG	NC	MSSA	N
**2213**	18/White	Knee	85/87	<95/<95	NG	289/-/0	MSSA	MSSA	MSSA	CNS	N
**2250**	24/White	Foot	58/62	<95/<95	NG	72.25/36/0	MSSA	CNS	NC	MSSA	N
**2306**	22/White	Foot	25/25	<95/<95	NG	38.25/0/0	CNS	CNS	NC	CNS	N
**2326**	23/White	Foot	**226**/**229**	100/289	NG	135/24/0	MSSA	MSSA	CNS	CNS	N
**2367**	20/White	Knee	**247**/**259**	<95/<95	NG	532/49/0	MSSA	MSSA	MSSA	MSSA	N
**2423**	21/White	Foot	92/86	103/<95	NG	36/30/0	NC	NC	NC	NC	N
**2637**	24/White	Elbow	10/12	<95/<95	NG	144/0/0	MSSA	MSSA	CNS	MSSA	N
**2758**	24/White	Arm, Elbow	13/<6	<95/<95	NG	-	MRSA	MRSA	MRSA	MRSA	N
**2867**	18/White	Thigh	147/138	<95/<95	NG	100/36/0	MSSA	MSSA	NC	NC	N
**2913**	18/White	Thigh	19/16	<95/<95	NG	90/0/0	CNS	CNS	CNS	CNS	N

Abbreviations and symbols: ND, not determined; NG, no growth; N, no; Y, yes; CNS, Coagulase-negative *Staphylococcus*; NC, not consented; MSSA, methicillin-sensitive *S*. *aureus*; MRSA, methicillin-resistant *S*. *aureus*

^a^ ASO and DNase B titers are represented as IU/ml and U/ml, respectively. Normal ranges: ASO <200 IU/ml, DNase B <301 U/ml. Titers were measured upon initial admission to the TMC (acute) and at 21–28 days post admission (conv). Elevated titer levels are depicted in bold.

^b^ Area measured in centimeters. A dash indicates no measurement taken.

### Clinical characteristics and risk factor assessment

For the risk factor assessment participants, most abscesses were caused by *S*. *aureus* (70.9%), 44.7% and 26.2% of which were MRSA and MSSA, respectively. PFT USA300 was the predominant *S*. aureus type (81.5%). Other observed pulsed-field types included USA400 (3.1%), and USA800 (1.5%). Infection site data for the 201 SSTI risk factor participants demonstrated anatomic differences between SSTI entities: cellulitis was more commonly observed on the lower extremities than abscesses (P<0.001) (Table 1).

Nasal *S*. *aureus* colonization was common and similar between both groups (52.4% and 51%). Additionally, colonization at any anatomic site was remarkably high − present in 91.3% of abscess participants and 86.7% of nonpurulent cellulitis participants. Interestingly, 62.8% of abscess patients were colonized with MRSA compared with 27.1% of cellulitis participants (P<0.001). With regard to potentially modifiable risk factors, cellulitis was associated with lower extremity blisters (P = 0.01).

### Culture analysis for microbiome participants

Culture data was available for 9 of the microbiome participants with cutaneous abscess. Of these 9, 5 were positive for MRSA (55.6%), 1 for MSSA (11.1%), and 3 grew non-*S*. *aureus* bacteria (33.3%) (Table 2). Unlike the cutaneous abscesses, only one of the 22 cellulitis aspirates analyzed (4.5%) grew bacteria (CNS) (Table 3). Body site culture analysis for all 40 microbiome participants revealed that *S*. *aureus* (MRSA and MSSA) was routinely found throughout the body (abscess group consented body sites: nose-52.9%, oropharynx-37.5%, perianal-33.3%, inguinal-20%) (cellulitis group consented body sites: nose-71.4%, oropharynx-42.9%, perianal-53.3%, inguinal-47.4%) (Tables 2 and 3). While MSSA and CNS were frequently isolated from all body sites, MRSA was only detected 6 times total: twice in the nose, once in the oropharynx, twice in the perianal region, and once at the inguinal body site. Of note, one cellulitis patient (ID# 2758) had MRSA cultured from all 4 body sites tested.

### ASO and anti-DNase B titer analysis

Despite the fact that they are not typically able to be cultured, *S*. *aureus* or streptococci are often assumed to be the cause of cellulitis. Therefore, we also assessed ASO and anti-DNase B titers in the cellulitis patients. At initial presentation, 7 of the 21 examined cellulitis patients (33.3%) had elevated ASO titers (Table 3). Of those 7 patients, 5 maintained elevated ASO titers up to 21–28 days post SSTI diagnosis. Only one participant who presented with normal ASO levels eventually increased to above normal levels at 21–28 days. Anti-DNase B titers were unremarkable for all cellulitis patients at both acute and convalescent time points. Of note, linear regression analysis revealed no correlation between ASO titer levels and *Streptococcus* 16S read (discussed below) abundance (data not shown).

### Sequencing results

In total, we sequenced 40 SSTI samples (18 purulent abscess and 22 cellulitis) in a single 454 pyrosequencing run that yielded 1,515,532 raw sequences. 562,027 (37.1%) reads remained after quality filtering and contaminant/chimera detection. Overall, our sample set contained 1,007 unique reads with an average read length of 257 (range, 230 to 283) nucleotides. On average, there were 30,744 reads associated with each purulent abscess sample (range, 6,663 to 55,430). However, we observed a reduction in associated reads for the cellulitis samples; 4 of the 22 cellulitis samples had 0 associated reads. For the remaining 18, there were on average 480 associated reads per sample (range, 149 to 956). This observation suggests that the bacterial load within the cellulitis samples is substantially reduced compared to the purulent samples. Good’s coverage values suggests that the number of reads associated with each sample accurately depict the total level of biodiversity (abscess samples: >99.4%; cellulitis samples: >95.9%). The percent abundance of each observed taxon for both infection types is included in S1 File.

### Purulent abscess microbiome

**Fig 1:**
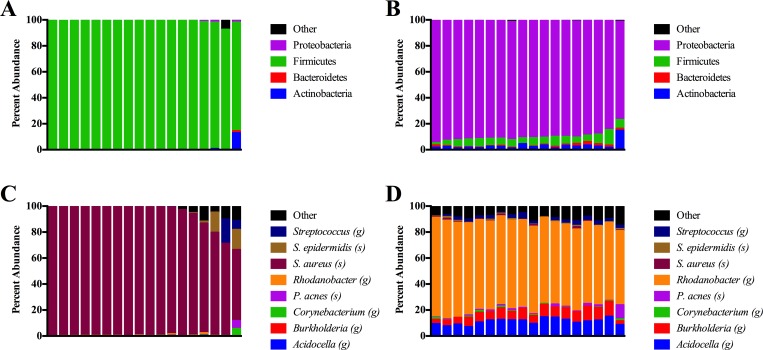
**Microbial composition within purulent abscesses (A and C) and cellulitis (B and D) samples.** The microbiomes were characterized at the bacterial phylum level (A and B) as well as genus (g) and species (s) levels (C and D). Each column corresponds to a single SSTI microbiome.

Analysis of the 18 purulent abscess samples revealed that the most abundant bacterial phylum present was Firmicutes (98.2% average abundance) (Fig 1A). At the species level, the most abundant bacterium present was *Staphylococcus aureus* (92.9% average abundance) (Fig 1C). Similar to a previous study [18], we observed a significant number of mixed infections; approximately 22% of the samples (4 of 18) had no single bacterial species reach an abundance level greater than 90%. While all purulent samples were sequence positive for *S*. *aureus*, additional abscess cohabitants included *Staphylococcus epidermidis*, *Streptococcus agalactiae*, *Staphylococcus haemolyticus*, and *Staphylococcus lugdunensis*.

### Cellulitis microbiome

Of the leading edge aspirate samples, 18 yielded usable sequences. In contrast to the purulent abscess samples, the most abundant bacterial phylum observed was Proteobacteria (89.8% average abundance) with Firmicutes representing the next most abundant phylum (5.9% average abundance) (Fig 1B). Further taxonomic investigation revealed that the most abundant bacterial species present was the gammaproteobacterium *Rhodanobacter terrae* (66.8% average abundance). While *Rhodanobacter* dominated the cellulitis samples, other clinically relevant genera present included *Streptococcus*, *Staphylococcus*, *Propionibacterium*, and *Burkholderia* (Fig 1D).

To confirm and extend the taxonomy observation obtained by looking at the V1-V3 portion of the 16S rRNA, we additionally amplified, cloned and sequenced at least 10 full-length 16S genes from four randomly chosen cellulitis samples: 2055, 2080, 2203, and 2250. In support of the 454 sequencing data, a large proportion of the full-length 16S sequences were classified as *Rhodanobacter* (Table 4). In addition, many of the sequences were assigned to the closely related *Dyella* genus. Besides *Rhodanobacter* and *Dyella*, we also detected *Acinetobacter* and *Streptococcus*.

**Table 4:** Full-length 16S rRNA taxonomy from cellulitis samples.

	2055	2080	2203	2250
**Clone 1**	*Rhodanobacter terrae*; SPg; FJ405366 100%	uncultured bacterium; nbu286c01c1; KF063132 100%	uncultured bacterium; nbu286c01c1; KF063132 98.8%	uncultured bacterium; nbu286c01c1; KF063132 100%
	*Rhodanobacter glycinis*; MO64; EU912469 99.8%	*Dyella sp*. K4; FR874237 99.1%	*Dyella sp*. K4; FR874237 94.2%	*Dyella sp*. K4; FR874237 99.1%
	*Rhodanobacter glycinis*; MO64; EU912469 99.8%	*Rhodanobacter sp*. A2-60; KF441591 98.4%	*Rhodanobacter sp*. A2-60; KF441591 90.3%	*Rhodanobacter sp*. A2-60; KF441591 98.4%
**Clone 2**	*Rhodanobacter terrae*; SPg; FJ405366 100%	uncultured bacterium; nbu286c01c1; KF063132 99.0%	uncultured bacterium; nbu286c01c1; KF063132 99.0%	uncultured bacterium; nbu286c01c1; KF063132 99.8%
	*Rhodanobacter glycinis*; MO64; EU912469 99.8%	*Dyella sp*. K4; FR874237 99.0%	*Dyella sp*. K4; FR874237 0.939	*Dyella sp*. K4; FR874237 0.989
	*Rhodanobacter terrae* str. SPg 96.42%	*Rhodanobacter sp*. A2-60; KF441591 98.3%	*Rhodanobacter sp*. A2-60; KF441591 90.1%	*Rhodanobacter sp*. A2-60; KF441591 98.3%
**Clone 3**	uncultured bacterium; nbu286c01c1; KF063132 99.2%	uncultured bacterium; nbu286c01c1; KF063132 99.4%	uncultured bacterium; nbu286c01c1; KF063132 99.4%	uncultured bacterium; nbu286c01c1; KF063132 99.3%
	*Dyella sp*. K4; FR874237 94.2%	*Dyella sp*. K4; FR874237 94.2%	*Dyella sp*. K4; FR874237 94.3%	*Dyella sp*. K4; FR874237 94.4%
	*Rhodanobacter sp*. A2-60; KF441591 90.8%	*Rhodanobacter sp*. A2-60; KF441591 90.9%	*Rhodanobacter sp*. A2-60; KF441591 89.1%	*Rhodanobacter sp*. A2-60; KF441591 90.1%
**Clone 4**	uncultured bacterium; nbu286c01c1; KF063132 99.9%	uncultured bacterium; nbu286c01c1; KF063132 99.9%	uncultured bacterium; nbu286c01c1; KF063132 98.5%	uncultured bacterium; nbu286c01c1; KF063132 99.3%
	*Dyella sp*. K4; FR874237 0.943	*Dyella sp*. K4; FR874237 94.8%	*Dyella sp*. K4; FR874237 93.7%	*Dyella sp*. K4; FR874237 94.2%
	*Rhodanobacter sp*. A2-60; KF441591 90.6%	*Rhodanobacter sp*. A2-60; KF441591 91.1%	*Rhodanobacter sp*. A2-60; KF441591 90.0%	*Rhodanobacter sp*. A2-60; KF441591 90.1%
**Clone 5**	uncultured bacterium; nbu286c01c1; KF063132 99.9%	uncultured bacterium; nbu286c01c1; KF063132 99.1%	uncultured bacterium; nbu286c01c1; KF063132 99.4%	uncultured bacterium; nbu286c01c1; KF063132 99.1%
	*Dyella sp*. K4; FR874237 0.948	*Dyella sp*. K4; FR874237 94.2%	*Dyella sp*. K4; FR874237 94.2%	*Dyella sp*. K4; FR874237 94.0%
	*Rhodanobacter sp*. A2-60; KF441591 91.1%	*Rhodanobacter sp*. A2-60; KF441591 90.2%	*Rhodanobacter sp*. A2-60; KF441591 90.6%	*Rhodanobacter sp*. A2-60; KF441591 90.5%
**Clone 6**^**a**^	uncultured bacterium; nbu286c01c1; KF063132 99.2%	uncultured bacterium; nbu286c01c1; KF063132 99.0%	bacterium BM0247; JQ680693 100%	uncultured bacterium; nbu286c01c1; KF063132 99.5%
	*Dyella sp*. K4; FR874237 94.1%	*Dyella sp*. K4; FR874237 0.933	*Escherichia coli*; r47; JQ661027 99.8%	*Dyella sp*. K4; FR874237 94.3%
	*Rhodanobacter sp*. A2-60; KF441591 90.1%	*Rhodanobacter sp*. A2-60; KF441591 89.5%	*Escherichia coli*; H16; JN129456 99.8%	*Rhodanobacter sp*. A2-60; KF441591 90.6%
**Clone 7**	*Rhodanobacter glycinis*; MO64; EU912469 100%	uncultured bacterium; nbu286c01c1; KF063132 99.5%	*Rhodanobacter terrae*; SPg; FJ405366 100%	uncultured bacterium; nbu286c01c1; KF063132 99.3%
	*Rhodanobacter sp*. B64; EU194895 100%	*Dyella sp*. K4; FR874237 94.4%	*Rhodanobacter glycinis*; MO64; EU912469 99.8%	*Dyella sp*. K4; FR874237 94.2%
	*Rhodanobacter terrae*; SPg; FJ405366 99.8%	*Rhodanobacter sp*. A2-60; KF441591 90.7%	*Rhodanobacter sp*. B64; EU194895 99.5%	*Rhodanobacter sp*. A2-60; KF441591 90.3%
**Clone 8**^**a**^	*Escherichia coli*; r47; JQ661027 99.1%	uncultured bacterium; nbu286c01c1; KF063132 99.4%	uncultured bacterium; nbu286c01c1; KF063132 98.3%	Uncultured bacterium JF204832 100%
	uncultured organism; ELU0124-T310-S-NI_000496; HQ791715 99.1%	*Dyella sp*. K4; FR874237 94.2%	*Dyella sp*. K4; FR874237 93.3%	uncultured bacterium; ncd2350h08c1; JF205603 100%
	*Escherichia coli*; NBRC 12062; AB680228 99.1%	*Rhodanobacter sp*. A2-60; KF441591 90.6%	*Rhodanobacter sp*. A2-60; KF441591 89.9%	*Streptococcus oralis* ATCC 700233 99.6%
**Clone 9**	uncultured bacterium GQ096144 99.6%	uncultured bacterium; nbu286c01c1; KF063132 99.1%	uncultured bacterium; nbu286c01c1; KF063132 99.6%	uncultured bacterium; nbu286c01c1; KF063132 99.4%
	*Acinetobacter parvus* KJ806336 99.6%	*Dyella sp*. K4; FR874237 93.9%	*Dyella sp*. K4; FR874237 94.5%	*Dyella sp*. K4; FR874237 94.2%
	uncultured bacterium; nbw109h05c1; GQ007804 99.3%	*Rhodanobacter sp*. A2-60; KF441591 90.4%	*Rhodanobacter sp*. A2-60; KF441591 90.9%	*Rhodanobacter sp*. A2-60; KF441591 90.6%
**Clone 10**	*Rhodanobacter glycinis*; MO64; EU912469 98.7%	uncultured bacterium; nbu286c01c1; KF063132 99.9%	uncultured bacterium; nbu286c01c1; KF063132 99.1%	uncultured bacterium; nbu286c01c1; KF063132 98.0%
	Rhodanobacter sp. B64; EU194895 98.7%	*Dyella sp*. K4; FR874237 94.8%	*Dyella sp*. K4; FR874237 93.9%	*Dyella sp*. K4; FR874237 93.2%
	*Rhodanobacter terrae*; SPg; FJ405366 98.0%	*Rhodanobacter sp*. A2-60; KF441591 91.1%	*Rhodanobacter sp*. A2-60; KF441591 88.8%	*Rhodanobacter sp*. A2-60; KF441591 90.0%
**Clone 11**	uncultured bacterium; nbu286c01c1; KF063132 99.9%		uncultured bacterium; nbu286c01c1; KF063132 99.5%	
	*Dyella sp*. K4; FR874237 94.8%		*Dyella sp*. K4; FR874237 94.6%	
	*Rhodanobacter sp*. A2-60; KF441591 91.1%		*Rhodanobacter sp*. A2-60; KF441591 90.9%	

Taxonomy information according to the RDP database. GenBank accession listed after species name. Top three hits per clone are given. Percentages represent percent identity.

^a^ *Escherichia coli* reads likely indicate purification of contaminate DNA from the *E*. *coli* TOP 10 cells that carried the cloned 16S sequences.

## Discussion

We used multiple current technologies and epidemiological data to investigate the etiology and concomitant risks factors for SSTI in a military population. With high-throughput sequencing and culture data, we confirmed that *S*. *aureus* is associated with cutaneous abscesses [18]. For nonpurulent cellulitis, however, we made a novel observation. Although small quantities of both streptococci and *S*. *aureus* sequences were recovered from most leading edge aspirate specimens, the most abundant species was the soil bacterium *Rhodanobacter terrae*. To our knowledge, this is the first clinical investigation that utilizes an epidemiological approach as well as standard microbiology, serology, and high-throughput sequencing to characterize cutaneous abscesses and nonpurulent cellulitis.

In the 18 microbiome participants with abscesses, we observed that *S*. *aureus* dominated most abscesses; however, there were 4 mixed infections. These findings build upon other work by our group. We recently characterized 40 abscess microbiomes in military trainees [18]. We found that while most abscess samples were dominated by *S*. *aureus*, polymicrobial abscess samples were prevalent. Given that polymicrobial SSTI are typically associated with severe disease such as necrotizing fasciitis [26, 27], it is surprising to find them in uncomplicated SSTI. Future studies should explore the pathogenesis of polymicrobial abscess as compared to monomicrobial infections.

Unlike abscesses, the most abundant bacteria detected in the nonpurulent cellulitis microbiomes were Proteobacteria, particularly from the *Rhodanobacter/Dyella* genera. While there are no reports that suggest that *Rhodanobacter* or *Dyella* infect humans or carry virulence factors, we can hypothesize that cellulitis pathology may be an immune response to the presence of these atypical bacteria. The role of the immune response in the pathology of cellulitis is further supported by previous reports that suggest an inflammatory component to cellulitis infections [28] and the observation that cellulitis more quickly resolves with the addition of NSAIDS and steroids [28, 29]. Furthermore, bacterial culture of cellulitis has been historically unsuccessful; a pathogen is isolated well less than 30% of the time [12–17]. Similarly, in our study only 1 of 22 leading edge aspirates yielded a bacterium (CNS). Thus, perhaps nonpurulent cellulitis is not caused by a traditional infection.

Similar to our study, a recent investigation also subjected needle aspirates to high-throughput sequencing [30]. These authors also identified atypical bacteria. However, they concluded that pyrosequencing/PCR may not be a suitable way to identify the etiology of cellulitis. We would propose a different interpretation of that study; perhaps the presence of these atypical bacteria causes cellulitis not due to active infection, but simply by invoking an active immune response. This possibility has yet to be confirmed but should be investigated further in the future. While the atypical bacteria observed in the Crisp *et al*. study (eg. *Acidovorax*, *Enterococcus*, and *Lactococcus*) were not seen in our study, this discrepancy may be explained by differences in study populations (U.S. emergency department patients versus military trainees), site of needle aspiration (center versus leading edge), sample transportation (anaerobic media versus -80°C), or sequencing data analysis pipelines (custom C# program versus mothur). Despite these differences, both studies strongly suggest a potential role of atypical bacteria in the etiology of cellulitis and together support the hypothesis that many cases of nonpurulent cellulitis may represent a nonspecific immune response to the presence of atypical bacteria.

Numerous reports have investigated the potential use of serology to aid in determining cellulitis etiology. Most of these serological tests (e.g. ASO) have aimed at detecting antibodies to BHS, given their role as a possible cause of cellulitis [11, 15, 16, 31, 32]. Indeed, 8 of the cellulitis patients in our study had elevated ASO titers at the acute and/or convalescent time points: a 36.4% seroconversion rate indicative of BHS exposure. In an investigation assessing serologic conversion, 73% of nonpurulent cellulitis was suggested to be caused by BHS and 27% was not identifiable [31]. Another study yielded a similar seroconversion rate (74%) for cellulitis/erysipelas [33]. Together those reports suggest the bulk of nonpurulent cellulitis may be caused by BHS. In contrast, while a substantial proportion of nonpurulent cellulitis participants were seropositive for BHS, our pyrosequencing data revealed that *Streptococcus* represented only a minor fraction of the total aspirate microbiota (approximately 2–5% average abundance) and was greatly outnumbered by the atypical bacteria discussed above (Fig 1D). Thus, these results challenge the current dogma that cellulitis is caused solely by *S*. *aureus* and beta-hemolytic streptococci.

We noted similarly high colonization rates between abscess and nonpurulent cellulitis participants, where nearly 90% were colonized with *S*. *aureus*. Additionally, as has been described by others, colonization prevalence was nearly doubled by assessing extra-nasal sites [34]. This colonization prevalence is similar to what Albrecht *et al*. found among 147 patients with abscesses, where 81% overall were colonized with *S*. *aureus* [35]. We also observed that 63% of abscess participants were colonized with MRSA and that both nasal and extra-nasal MRSA colonization was associated with abscesses. This MRSA colonization prevalence is similar to the 61% described by Fritz et al. in a pediatric population [36]. Taken together, these data lend further support to the role of colonization in the pathogenesis of purulent SSTI.

We observed that compared to abscesses, nonpurulent cellulitis more frequently affected the lower extremities, and was associated with blisters. The lower extremity has been described as the most common site for cellulitis in both military and non-military populations [37, 38]. Skin disruption is a risk factor that has been described for the development of lower extremity cellulitis, and blisters are especially common early in military training [38, 39]. It may be that skin disruption permits increased localized bacterial burden, as has been described with tinea pedis [40].

There are clear limitations to our study. First, leading edge aspiration was performed only on cellulitis participants and not on unaffected skin. Unfortunately, we were unable to receive IRB approval to obtain aspirates from active duty military personnel who were not suffering from cellulitis. It should be noted that a few studies have begun to characterize the healthy subcutaneous tissue microbiome, and have detected bacteria such as *Propionibacterium acnes*, *Staphylococcus epidermidis*, and *Pseudomonas spp*. in both the reticular and dermal adipose layers [41]. Interestingly, all three of these organisms were also detected in our cellulitis aspirates at very low levels (S1 File). One may only speculate on the role of these subcutaneous bacteria in health or in disease. Secondly, we assessed colonization and potential risk factors only at presentation and not at the beginning of training; therefore, we cannot determine whether colonization or other risk factors preceded infection. Third, with regard to ASO titers, it is difficult to determine if these titers are associated with cellulitis or recent BHS infection prior to arrival. Alternatively, given the low abundance of bacteria observed in these uncomplicated and non-hospitalized cellulitis cases, it is also possible that ASO titers may not yet be detectable (false-negative). We also acknowledge that while ASO titers did not correlate with the *Streptococcus* 16S reads, those sequences are portrayed as relative abundance; thus, we are unable to assess total bacterial load at any body site. Lastly, our study population is composed entirely of young, healthy males in a setting where infections are actively monitored and prevented. Therefore, our results may not be generalizable to SSTIs in the general population or in immunocompromised patients.

In conclusion, our results emphasize that cutaneous abscess and nonpurulent cellulitis represent two distinct diseases. While purulent abscesses are rich in *S*. *aureus* and associated with MRSA nasal colonization, we detected atypical bacteria such as *Rhodanobacter/Dyella* in our cellulitis specimens. Thus, while both infections are SSTI and share overlapping clinical characteristics, they differ in etiology and likely in pathogenesis. Future work should seek to capitalize on advances in diagnostic and sequencing technologies as means to investigate these distinct disease states.

## Supporting Information

S1 File Percent abundance of each taxon per sample according to the GreenGenes database. Each column represents one sample and is labeled with sample ID, infection group (abscess or cellulitis), and total number of reads associated with each sample. The results for the PBS negative control are included.
(XLSX)
